# A detailed comparison between the endoscopic images using blue laser imaging and three-dimensional reconstructed pathological images of colonic lesions

**DOI:** 10.1371/journal.pone.0235279

**Published:** 2020-06-29

**Authors:** Takeshi Ueda, Kohei Morita, Fumikazu Koyama, Yuichi Teramura, Tadashi Nakagawa, Shinji Nakamura, Yayoi Matsumoto, Takashi Inoue, Takayuki Nakamoto, Yoshiyuki Sasaki, Hiroyuki Kuge, Maiko Takeda, Chiho Ohbayashi, Hisao Fujii, Masayuki Sho

**Affiliations:** 1 Department of Surgery, Nara Medical University, Kashihara, Japan; 2 Department of Surgery, Minami-Nara General Medical center, Yoshino, Nara, Japan; 3 Department of Diagnostic Pathology, Nara Medical University, Kashihara, Japan; 4 Department of Endoscopy, Nara Medical University Hospital, Kashihara, Japan; 5 Clinical Research Endoscopy System Division and Medical System Business Division, FUJIFILM Corporation, Tokyo, Japan; 6 Department of Surgery, Saiseikai Chuwa Hospital, Sakurai, Japan; 7 Department of Surgery, Takanohara Central Hospital, Nara, Japan; 8 Gastrointestinal Endoscopy and IBD center, Yoshida Hospital, Nara, Japan; Nicolaus Copernicus University, POLAND

## Abstract

Blue laser/light imaging (BLI) is an image-enhanced endoscopy (IEE) technique that can provide an accurate diagnosis by closely observing the surface structure of various colonic lesions. However, complete correspondence between endoscopic images and pathological images has not been demonstrated. The aim of this study was to accurately compare endoscopic images and the pathological images using a three-dimensionally (3D) reconstructed pathological model. Continuous thin layer sections were prepared from colonic tissue specimens and immunohistochemically stained for CD34 and CAM5.2. Three-dimensional reconstructed images were created by superimposing immunohistochemically stained pathological images. The endoscopic image with magnifying BLI was compared with the top view of the 3D reconstructed image to identify any one-to-one correspondence between the endoscopic images and histopathological images using the gland orifices and microvessels as a guide.

Using 3D reconstructed pathological images, we were able to identify the location on the endoscope image in cases of colonic adenocarcinoma, adenoma and normal mucosa. As a result, the horizontal plane of the endoscopic image and the vertical plane of the 2D pathological specimen were able to be compared, and we successfully determined the visible blood vessel depth and performed a detailed evaluation on magnifying BLI. Examples are as follows: (1) The median vasculature depth from the mucosal surface that could be recognized as vasculature on magnifying BLI was 29.4 μm. The median depth of unrecognizable vessels on magnifying BLI was 218.8 μm, which was significantly deeper than recognizable vessels. (2) Some brownish structures were suggested to potentially be not only dense vessels, vessel expansions, corrupted vessels but also bleeding or extravasation of erythrocytes. Overall, we demonstrated a new approach to matching endoscopic images and pathological findings using a 3D-reconstructed pathological model immunohistochemically stained for CD34 and CAM5.2. This approach may increase the overall understanding of endoscopic images and positively contribute to making more accurate endoscopic diagnoses.

## Introduction

In recent years, newly developed image-enhanced endoscopy (IEE) techniques, such as narrow banding imaging (NBI), blue laser/light imaging (BLI) and linked color imaging (LCI) have enabled the observation of the surface structure of various colonic lesions using narrow-band laser light [1–4]. In particular, NBI and BLI with magnification can predict the histopathological diagnosis and invasion depth with good diagnostic effectiveness [1–3]. These studies have shown that the patterning of endoscopic images is associated with an increased pathological diagnostic accuracy. Other studies have pathologically analyzed the microvessel count, diameter and depth from the surface in hyperplastic polyp or adenoma, and reflected these findings in endoscopic findings [5–7]. In these studies, the authors classified the endoscopic findings to predict the most invasive tumor depth or histological diagnosis, and what was observed in endoscopy was not necessarily directly reflected by the pathological findings.

However, it is difficult to accurately establish any correlation between endoscopic images and pathological images. To our knowledge, there have been no reports in which the endoscopic images and the pathological images showed complete one-to-one correspondence. The three-dimensional (3D) reconstruction of serial sections is useful for understanding the microstructure and microvasculature of the colonic tumor surface. Some studies have shown the 3D microvasculature of pre-cancerous lesions and invasive carcinomas of the colon, and 3D imaging may be used in combination with the standard histology to better characterize the tumor microenvironment in colorectal neoplasms [8–10]. The aim of this study was to compare the endoscopic findings and the pathological microstructures using the 3D reconstruction of microstructures and microvessels on the surface of colonic tumors to establish a one-to-one correspondence.

## Patients and methods

### The endoscopic examination and system

The LASEREO endoscope system (Fujifilm Co., Tokyo, Japan) uses semiconductor lasers as a light source [3,11]. It has two types of lasers with bandwidths of less than 2 nm, with center wavelengths of 410 nm and 450 nm. The 450 nm laser illuminates a phosphor to produce white light illumination similar to a conventional xenon lamp. The 410 nm laser is used to observe on the mucosal surface, including surface blood vessel and structure patterns. The BLI-bright mode, which combines strong 410nm laser light and weak 450 nm laser light is useful for observing the mucosal surface with brighter illumination. Endoscopic examinations were carried out wearing a cap, and using normal WL, magnifying BLI, and chromoendoscopy with indigo carmine. Magnifying BLI was performed using an EG-L590ZW endoscope (Fujifilm Co., Tokyo, Japan) with BLI-bright mode. Endoscopic images were evaluated using still imaging. The tumor shape was classified based on the Paris endoscopic classification of superficial neoplastic lesions in the digestive tract.

### Human specimens

**Fig 1:**
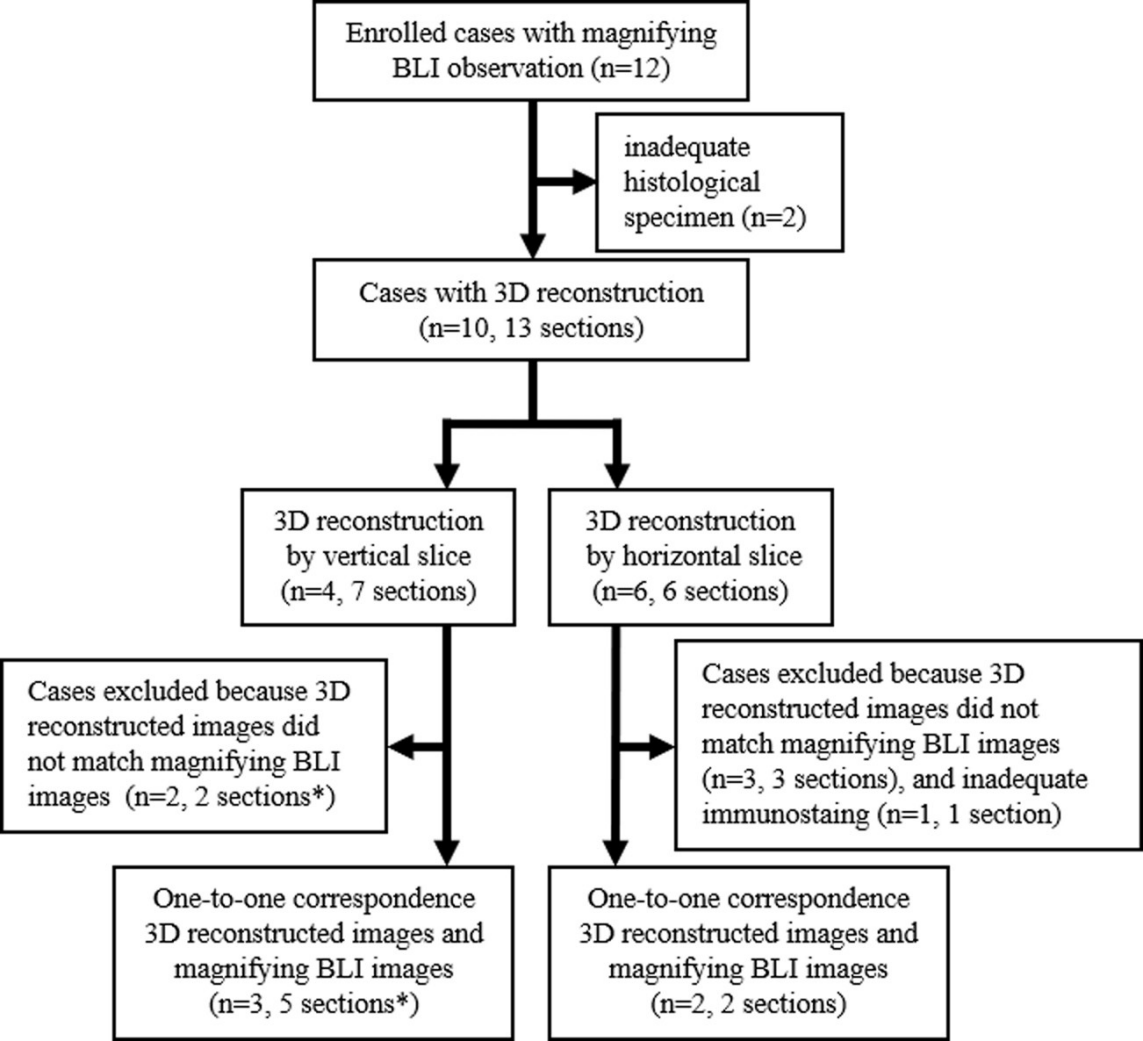
Enrolled patients flow chart. *In one patient, there were two sections with or without matching between 3D reconstructed images and images of magnifying BLI.

This study was approved by the Institutional Review Board of Nara Medical University (ethical approval No.831) and was carried out in accordance with the Declaration of Helsinki. Written consent was obtained from the patient for the use of the obtained tissue and participation for their in this study. Twelve patients were enrolled between July 2013 and June 2017 at Nara Medical University Hospital for inclusion in this study (Fig 1). Colonic lesions were obtained by endoscopic mucosal resection (EMR) or surgical resection.

### Pathological processing and immunohistochemistry

The resected specimens were subsequently fixed with 10% buffered formalin. The fixed specimens were divided perpendicularly to the mucosal surface with a width of 2mm and embedded in paraffin. We selected one of the divided specimens that had already been subjected to a pathological examination to make 100 continuous perpendicular or horizontal slices of 3 μm in thickness using an AS-400M (Kurabou Industries, Osaka, Japan).

Immunohistochemical staining was performed using the avidin-biotin-peroxidase complex method, an automated staining system (Bond-Ⅲ; Leica Biosystems, Tokyo, Japan), and antibodies against CD34 (QBEnd10; Dako Japan, Tokyo, Japan) and cytokeratin (CAM5.2; BD Biosciences, Tokyo, Japan), as markers of the vascular endothelium and mucosal epithelium, respectively. Immunohistochemical staining of CAM5.2 was performed every three slices; the other slices were subjected to CD34 immunohistochemical staining.

### Image processing and analyses

The positively stained parts were extracted from digital microscope images of each slide using the Photoshop CC software program (Adobe Systems Inc., San Jose, CA). Regarding the blood vessel reconstruction, the CD34-positive parts corresponding to the blood vessel wall were extracted and drawn as gray scale images. The inner area of the extracted positive part forming the circle that can be regarded as the inside of the blood vessel was also drawn as the positive part. Incompletely extracted blood vessels, such as the undetectable thin vessel walls, were considered to be positive regions. The CAM5.2 positive parts were used for reconstruction of the crypt glands. The CD34 images were converted to gray scale using different gray levels. Each image was aligned with the other images in consideration of continuity, then the adjacent CAM5.2 edited images and CD34 extracted images were superimposed. A set of continuous superimposed slide images was imported into the Synapse VINCENT (Fujifilm Co., Tokyo, Japan) software program, which is a 3D medical image analysis system already used in surgical simulations [12, 13], to reconstruct the 3D histopathological image. Thin slice images of the microvasculature 50, 100 and 150μm from the mucosal surface were also made from superimposed images to obtain a 3D reconstructed image of the microvasculature to validate the visible blood vessels on the endoscopic images.

The endoscopic images were compared with the top view of the 3D reconstructed image and gland orifices to find one-to-one correspondence between the endoscopic image and histopathology slide with consideration for the histological specimen’s position, slice direction, and expected distortion of the tissue. Sites of the 3D pathological images that were under consideration were observed in detail endoscopically and marked by coagulation. Surgically resected cases were marked with clips before surgery. Pathological specimens were created using the marking part as a guide, and 3D images were constructed. The endoscopic image and 3D reconstructed image were manually matched without the use of special equipment.

We conducted a validation test to verify the depth of the visible microvessels by magnifying BLI (case 4). The selected endoscopic image and the corresponding pathological image were divided into 50 areas. In the selected area, an endoscopist (T.U.) determined whether or not the blood vessels were visible in the divided section without evaluating the pathological image. A pathologist (M. K.) measured the depth of the nearest blood vessel from the surface in each of the divided areas by observing a CD34-immunostained slide using a microscope, without evaluating the endoscopic image. The depth of the microvessels was defined as the depth from the mucosal surface to the center of the vessel lumen. The statistical analysis was performed using the StatMate IV software program (Advanced Technology for Medicine and Science, Tokyo, Japan). The quantitative analysis was performed using the Mann-Whitney U test. P-values of <0.05 were considered to indicate statistical significance.

## Results

### 3D reconstructed histological images and one-to-one correspondence

Colonic specimens were obtained from 12 patients endoscopically and surgically. In two patients who underwent surgical resection, adequate continuous thin slices for 3D reconstruction could not be obtained after the pathological examination (both submucosal invasive cancer cases). Thirteen sections from 10 patients were used to create reconstructed 3D images. Seven sections from four patients were sliced perpendicular to the mucosal surface, and six sections from six patients were sliced horizontally. Five sections from four patients were excluded due to incomplete matching between the endoscopic images and 3D reconstructed images, and one section from one patient was excluded due to inadequate immunohistochemical staining.

Ultimately, seven colonic sections from five patients were analyzed in this study (Fig 1). The details of the five patients are summarized in Table 1. Two patients received colonic resection for adenocarcinoma and three patients received endoscopic mucosal resection for adenocarcinoma and tubular adenoma with low-grade atypia.

**Table 1:** Cases of one-to-one correspondence between endoscopic images and 3D pathological reconstructed images.

case	age	Sex	location	shape	size (mm)	resection	depth	section	histology	slice
1	59	M	S	0-Is	10×8	EMR	M	1-a	ADC	vertical
				normal (peritumor)				1-b	NM	vertical
2	70	M	S	2	15×15	operation	MP	2	ADC	horizontal
3	61	F	D	0-Is	20×18	operation	M	3	ADC	horizontal
4	65	M	S	0-Ip	7×7	EMR		4	LGA	vertical
5	52	M	S	normal (peritumor)		EMR		5-a	NM	vertical
				normal (peritumor)				5-b	NM	vertical

S: sigmoid colon, D: descending colon, 0-Is: sessile, 0-Ip: pedunculated, EMR: endoscopic mucosal resection, M: mucosal invasion, MP: muscular invasion, ADC: adenocarcinoma, LGA: low-grade adenoma, NM: normal mucosa

### Matching the endoscopic images and 3D reconstructed histological images

#### One-to-one correspondence in normal mucosa

The 3D reconstructed histological images using normal mucosa near the tumor were approximately 1.5 mm × 1.0 mm × 0.4 mm in size. The crypt orifices, which were round in shape, were of uniform size. The crypt glands stood vertically. Microvessels stood without a network between the crypts and became thin and uniform. The organization of microvessels had a consistent pattern, and the mucosal capillary network was arranged in a honeycomb pattern around the mucosal glands (S1 Video).

**Fig 2:**
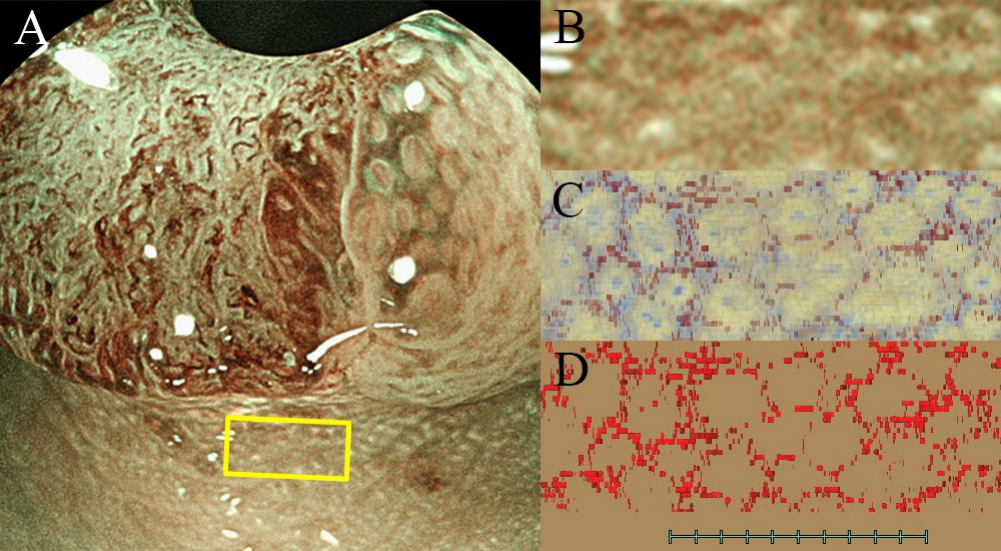
Matching between the endoscopic images and 3D reconstructed histological images in normal mucosa near the tumor (Section 1-b). (A) The yellow lines correspond to the suspicious area on the 3D reconstructed images. (B) Magnifying BLI. (C) Top view of the 3D image constructed by VINCENT from slides of multiple histopathological slices. (D) Top view of the 3D reconstructed vessels image.

Fig 2 shows the estimated corresponding area of magnifying BLI and the top view of the 3D reconstructed image. The array of the crypt orifices was correlated with that of the endoscopic image. The top view of the 3D reconstructed images of the blood vessels that were also extracted near the surface are shown in Fig 2. The image of a 3D reconstructed microvessel showed honeycomb structures associated with the brown lines on magnifying BLI. The endoscopic image and 3D reconstructed image were matched using the cut line as an index; however, it was extremely difficult to perform matching because the structure was uniform.

#### One-to-one correspondence in benign adenoma

Some of the crypts appeared to be extended and distorted. The crypt orifices, which were oval and tubular in shape, were of various sizes. Microvessels were standing vertically between the crypts and became thick and distorted. The capillary networks were only formed around the crypt orifices near the mucosal surface. (S2 Video).

**Fig 3:**
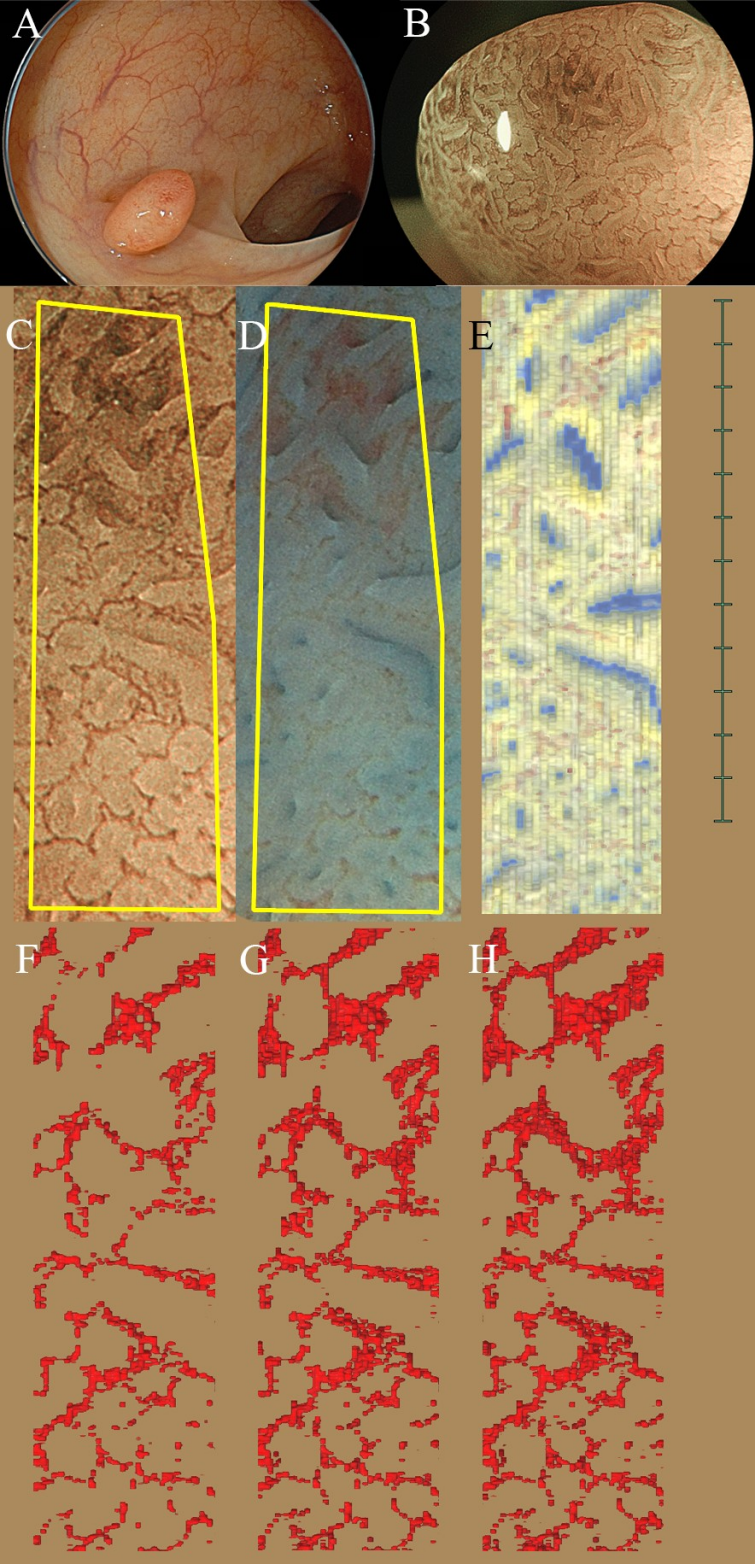
A complete match between the endoscopic images and 3D reconstructed histological images in colonic adenoma (Section 4). (A) Non-magnifying WL image. (B) Magnifying BLI. (C, D) The yellow lines correspond to the suspicious area on 3D reconstructed images. C: Magnifying BLI. D: Chromoendoscopic image stained by indigo carmine. (E) Top view of the 3D reconstruction images immunohistochemically stained for CD34 and CAM5.2. (F-H) Top view of the 3D vessels image extracted from the surface. (F) 50μm. (G) 100μm. (H) 150 μm thickness.

Fig 3 shows the estimated corresponding area of magnifying BLI, the chromoendoscopic image, and the top view of the 3D reconstructed image. The corresponding area of the 3D reconstructed image on the endoscopic images was recognized as a distorted rectangle (Fig 3C and 3D). Although there was some distortion due to the difference in the distance of the endoscope from the mucosal surface, the difference in extensibility by the endoscopic lens and the transformation distortion of the tissue during the histological slide-making process, the pattern of the crypt orifice was well correlated with that of the chromoendoscopic image. The top view of the 3D reconstructed images of the blood vessels that were also extracted near the surface (thickness, 50, 100 and 150 μm) are shown in Fig 3 (Fig 3F–3H). The image of each 3D reconstructed microvessel showed meshed and condensed structures associated with the brown lines on magnifying BLI. Based on these crypt and blood vessel patterns, the position of the histological images and the endoscopic images were considered to show one-to-one correspondence.

#### One-to-one correspondence in adenocarcinoma

**Fig 4:**
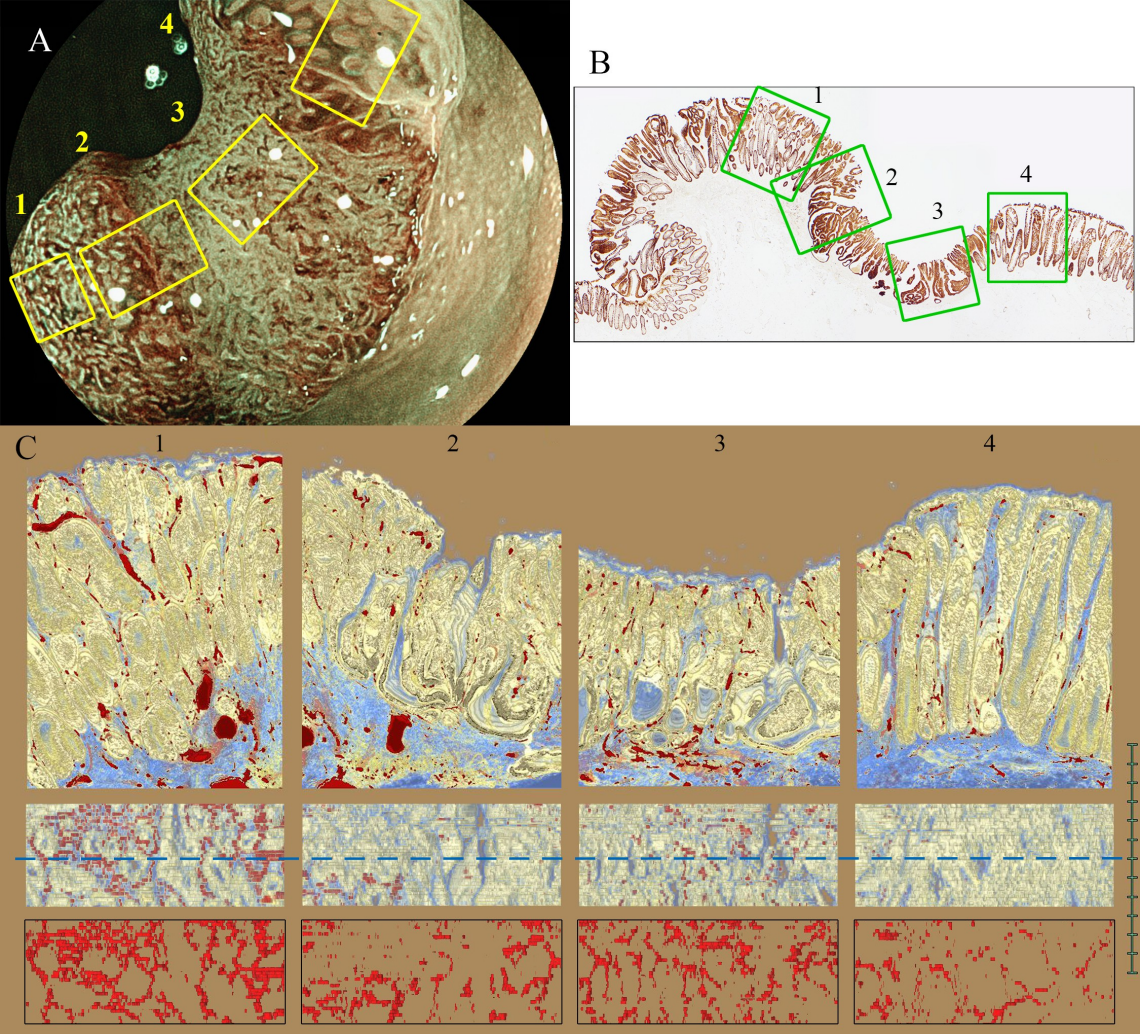
Matching between the endoscopic images and 3D reconstructed histological images in mucosal invasive adenocarcinoma (Section 1-a). (A) Magnifying BLI. The yellow lines correspond to the suspicious area on 3D reconstructed images. (B) The 2D pathological section of the 3D reconstructed region. (1–4) First row: The cross-sectional view of the 3D reconstruction images shows that some of the crypts appeared to be distorted and were of varying size. Second row: Top view of the 3D reconstructed image immunohistochemically stained for CD34 and CAM5.2. The blue lines correspond to the cross-sectional views (first row). Third row: Top view of the 3D reconstructed vessel images. The image of each 3D reconstructed microvessel showed distended and condensed structures.

In the tumor sections of case 1 (1-a section), four 3D images containing adenocarcinoma were reconstructed (Fig 4 and S3–S6 Videos). In all four 3D images, we were able to confirm crypts and microvessels. Some of the crypts appeared to be distorted irregularly. The crypt orifices were of various sizes. Microvessels stood vertically between the crypts and became thick and distorted. The capillary networks were irregular in diameter and formed only around the crypt orifices near the mucosal surface of the adenocarcinoma area. The top view of the 3D reconstructed images of the microvessels was drawn (thickness, 100 μm). The image of each 3D reconstructed microvessel showed distended and condensed structures associated with magnifying BLI.

**Fig 5:**
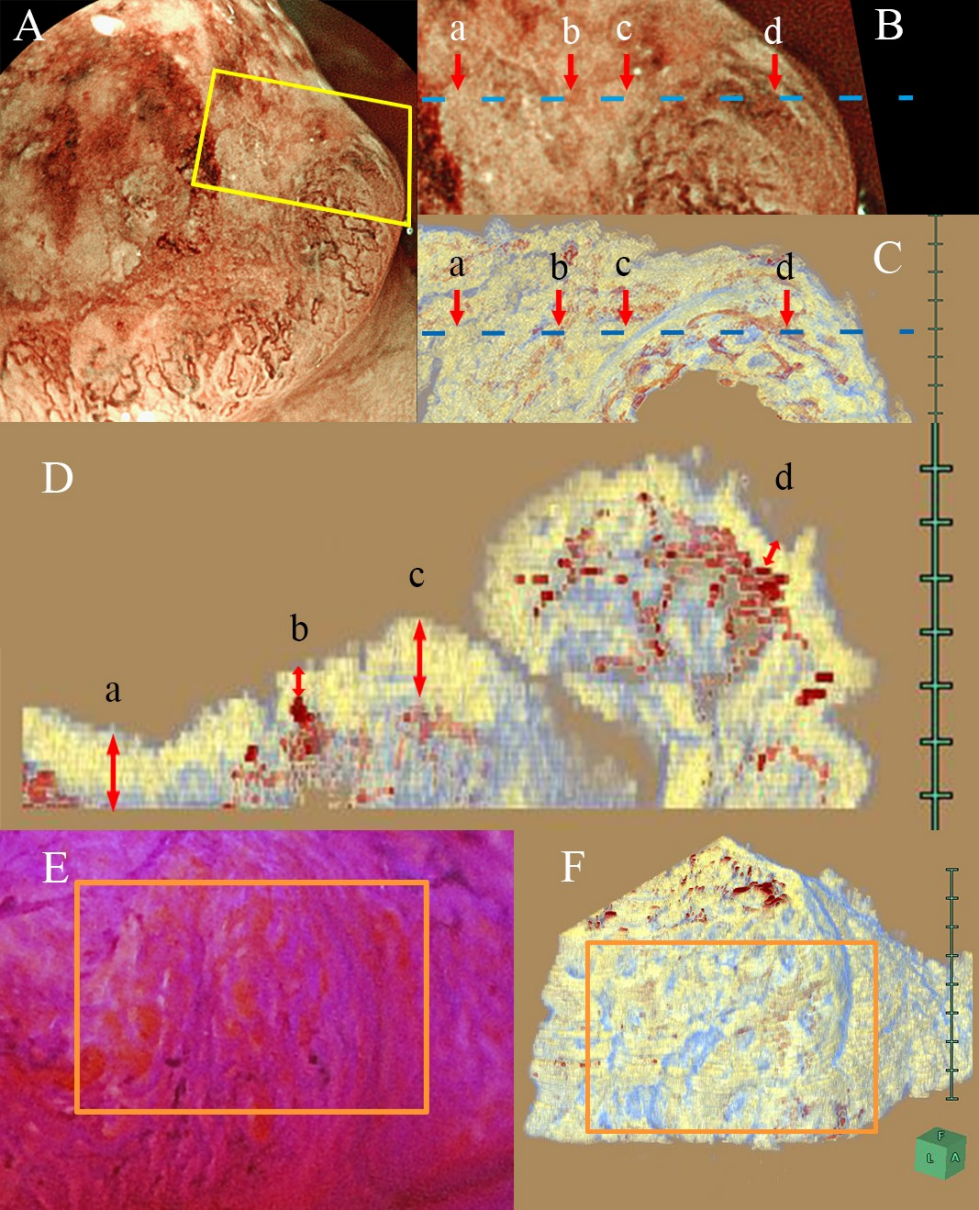
Matched images between the endoscopic images and 3D reconstructed histological images in muscle invasive adenocarcinoma (Section 2). (A) An overview of the tumor. The yellow lines correspond to the suspicious area on 3D reconstructed images. (B) Magnifying BLI of matching region. (C) Top view of 3D reconstruction image. (D) Cross-sectional view of the matching region (blue line in B and C). The depth of distended vessels detected by magnifying BLI was 30 and 50 μm, but the microvessels not detected by magnifying BLI were located more deeply, at 120 or 180 μm. (a) 120 μm, (b) 50 μm, (c) 180 μm and (d) 30 μm. (E) Endoscopic image stained by crystal violet. The orange lines correspond to the suspicious area on the 3D reconstructed image. (F) 3D reconstructed image.

Case 2 was a type 2 tumor with muscle layer invasion, and a 3D pathological image was reconstructed using horizontal slices at the marginal part (S7 Video). In the top view of the tumor, there were unclear crypt orifices, few microvessels and non-vessel areas at the surface of the depression part, and distended microvessels were noted at the surface of the perimeter part. We matched image of magnifying BLI and the top view of the 3D reconstructed image using these distended vessels as an index (Fig 5B and 5C). The 2D reconstructed cross-sectional vertical view showed that the depth of the distended vessels detected by magnifying BLI was 30 and 50 μm, but the microvessels not detected by magnifying BLI were located more deeply at 120 or 180 μm (Fig 5D). The side wall of the same 3D reconstructed image had stretched gland orifices that matched an endoscopic image stained with crystal violet (Fig 5E and 5F). Endoscopic images and 3D reconstructed images were able to be matched using the characteristic parts of the surface image in all adenocarcinoma cases.

### Matching the endoscopic images and 2D pathological sections reflecting 3D reconstructed histological images

When the 3D reconstructed images and endoscopic images were found to correspond, it was assumed that the further comparison of endoscopic images and pathological findings could be performed. In the detailed analysis of the surface layer including the depth of the microvessels and microstructures, we needed to perform matching at a scale of a few micrometers in order to compare endoscopic images and 2D vertical pathological sections. This was difficult in specimens that had been 3D reconstructed using horizontal slices or pathological specimens with a poor surface condition. Therefore, we selected an adenoma case (case 4), which showed the best matching, for the comparison of magnifying BLI and 2D pathological sections.

#### The analysis of the depth of visible microvessels from the mucosal surface

**Fig 6:**
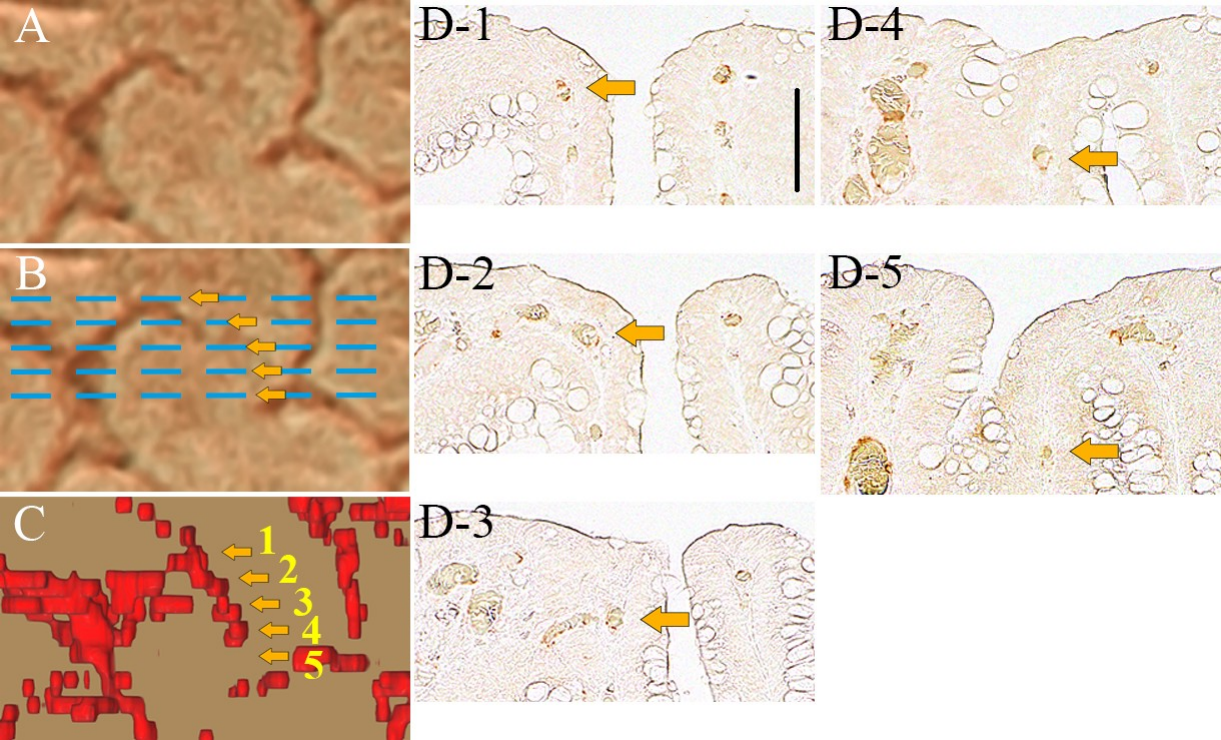
One of visible blood vessels that disappeared on magnifying BLI. (A, B) Magnifying BLI. Blue lines are pathological histopathological section lines. (C) Top view of the 3D reconstructed vessels extracted from surface (50 μm). The arrows correspond to the vessels in each pathological section. (D) CD34 stained histopathological images corresponding to the blue line in B. Arrows indicate the corresponding vessel. Black bar indicates 100 μm.

We focused on one of visible blood vessels that disappeared on magnifying BLI, and the corresponding blood vessel running from near the mucosal surface to the deep area could be tracked on the adjacent histopathological images (Fig 6). The depths of the target blood vessel visible on magnifying BLI were 28.8 μm, 27.3 μm, and 44.0 μm from the mucosal surface, respectively (Fig 6D1–6D3). On the other hand, the depths of the blood vessels in the invisible areas on magnifying BLI were 60.6 μm and 80.0 μm, respectively (Fig 6D4–6D5).

**Fig 7:**
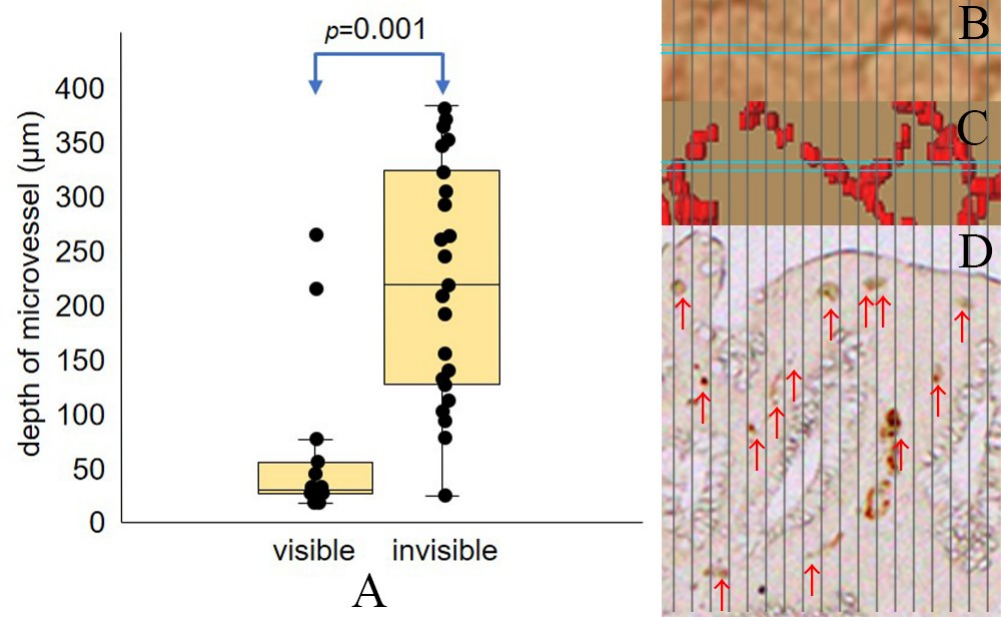
The assessment of the depth at which micro vessels were visible. (A) The median visible vascular depth was 29.4μm and the median invisible vascular depth was 237.9μm. There was a significant difference between the two groups (p = 0.001). (B-D) A schematic illustration of the test of the depth from the mucosal surface at which microvessels were visible. (B) Magnifying BLI in which the endoscopist assessed the visibility of the microvessels in the blue box. (C) The 3D reconstructed image used for the alignment. (D) CD34 stained histopathological image for which the pathologist measured the depth of the vessels indicated by red arrows. Vertical black lines indicate the segmentation.

The validation test of the vascular depth was conducted using another corresponding area. The test line was 0.86 mm long, and the vasculature of up to 400 μm in depth on the histopathological slide was measured. The median depth from the mucosal surface at which vasculature could be recognized on magnifying BLI was 29.4 μm (17.5–266.2 μm). The median depth at which vasculature could not be recognized was 218.8 μm (23.6–382.7 μm). There was a significant difference between the two groups (p = 0.001) (Fig 7).

#### Some brownish structures

**Fig 8:**
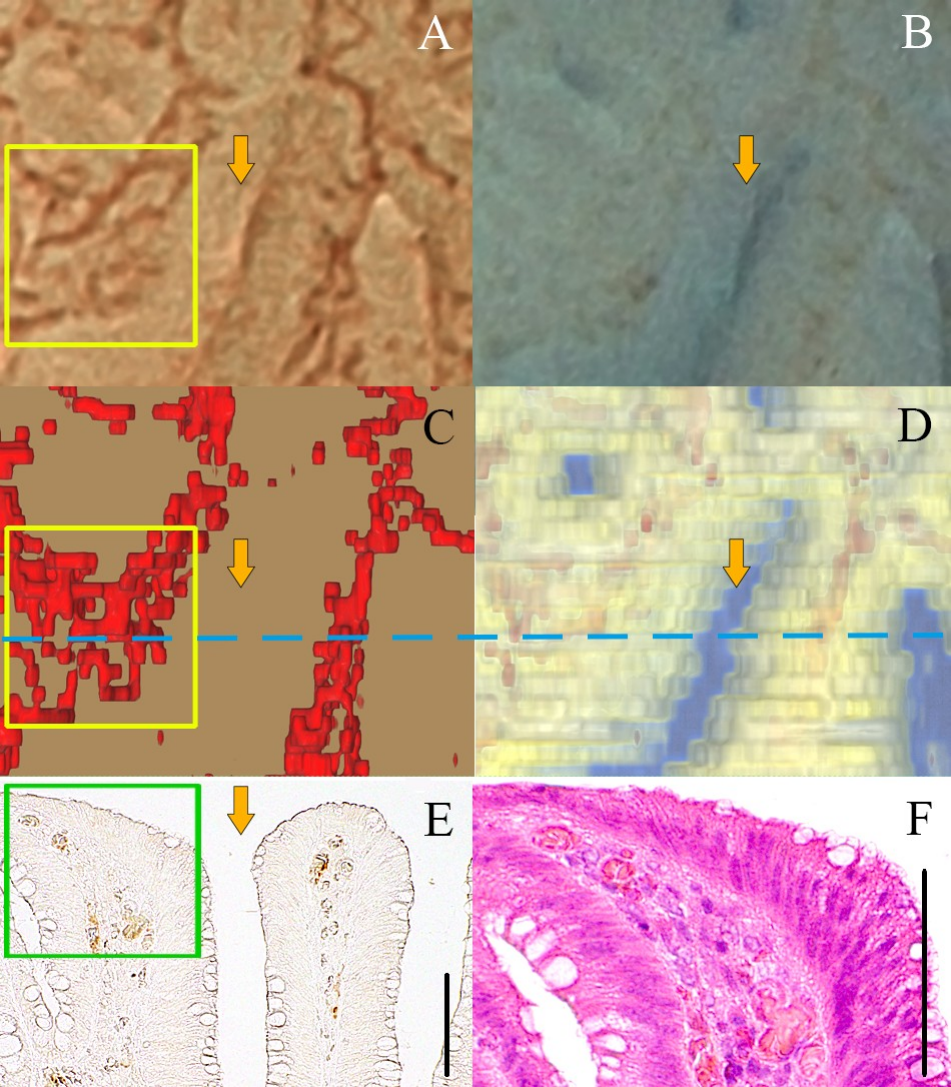
Light brownish areas and the gland orifice recognized as a brown line. (A) Magnifying BLI. (B) Chromoendoscopic image stained by indigo carmine. (C-D) Top view of the 3D reconstructed vessels extracted from surface with 50 μm, and histopathological tissue. (E) CD34-stained histopathological image of the same position. (F) Hematoxylin eosin re-stained histopathological image in the green box of (E). Arrows indicate the corresponding pit. Black bars in (E) and (F) indicates 100 μm.

Some of the open crypt orifices confirmed by the chromoendoscopy and the 3D reconstructed image were also detected as brown lines similar to blood vessels on magnifying BLI (Fig 8A–8E). It was also confirmed that a similar vascular pattern was not detected in the corresponding area by the 3D reconstructed microvessel images. Some of them were accompanied by a bright line that was not observed at the location of the brown lines corresponding to the blood vessels.

**Fig 9:**
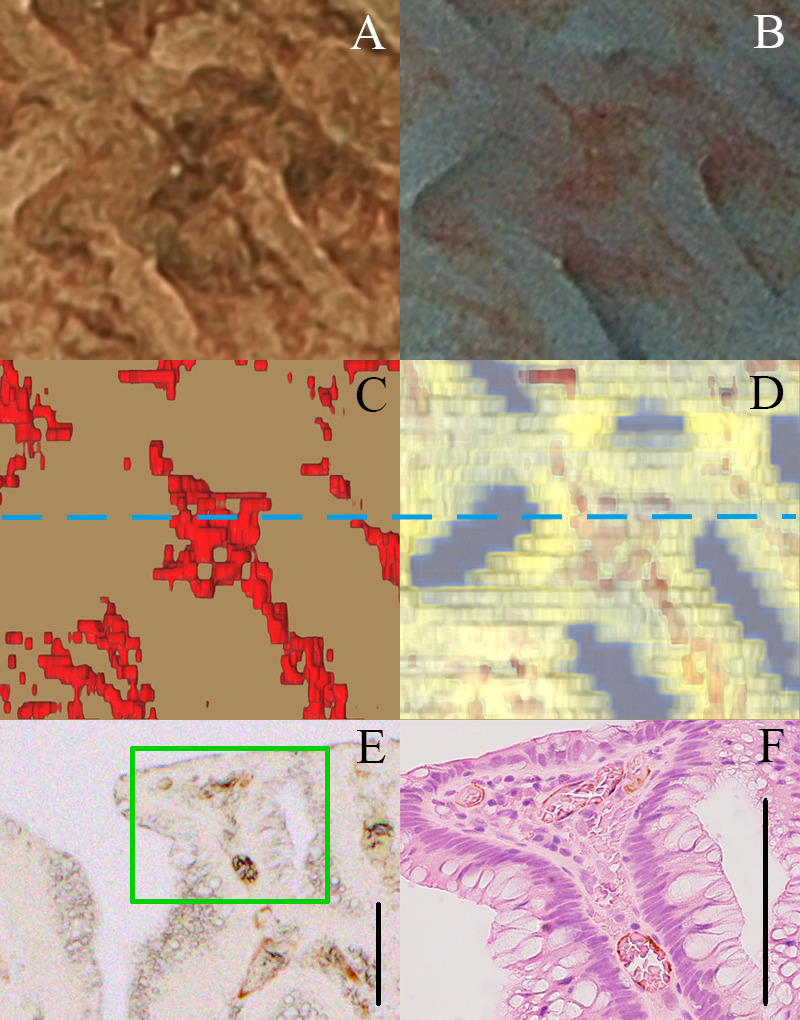
Dark brownish area. (A) Magnifying BLI. (B) Chromoendoscopic image stained by indigo carmine. (C-D) Top view of the 3D reconstructed vessels extracted 50μm from the surface, and histological tissue. (E) CD34-stained histopathological image (blue line in (C) and (D)). (F) Hematoxylin eosin re-stained histopathological image (green box of (E)). Black bars in (E) and (F) indicates 100 μm.

It was also possible to detect some brownish areas on magnifying BLI. Some of the brownish areas appeared light, while others appeared dark. In the light brownish areas, small network formation of capillary vessels at the mucosal surface was recognized not only on the magnifying BLI but also in the histopathological findings (Fig 9A–9F). In the dark brownish area where a homogenous brown color was observed on magnifying BLI, a reddish area was observed on the chromoendoscopic image, and the concentration of the blood vessels was observed on the 3D reconstructed microvessel image (Figs 9A–9D). The histopathological findings included vessel expansions, corrupted vessels and extravasation of erythrocytes (Fig 9E and 9F).

## Discussion

The current study is the first to describe that the histopathological images were precisely matched with the endoscopic findings using a 3D histopathological reconstruction model. It is extremely difficult to completely match magnifying endoscopic findings and the histopathological findings because it is difficult to understand the correspondence between the surface feature of endoscopic imaging and vertical sectional view of the histopathological images, which have a slice interval of millimeters. In this study, we demonstrated that the 3D reconstructed image of the microscopic multi-slice images immunohistochemically stained with CAM5.2 (mucosal epithelium marker) and CD 34 (vascular endothelium marker) made it possible to compare the top view of the tissue and the endoscopic image. Once the 3D reconstructed images and endoscopic images could be matched, the direction and position of the histopathological image was clarified on the endoscopic image, so we could compare the objects on the endoscopic images and the pathological findings in detail. The current study revealed that the microstructure observed on magnifying BLI was a few micrometers in scale in both the horizontal and the vertical directions, and visible microvessels were in the extremely superficial layer from the mucosal surface in colonic lesions.

It is well known that IEE specialized in imaging microvascular systems such as NBI and BLI is useful for diagnosing the depth of invasion. Its usefulness for treatment decision making has been increased by patterning and it is now frequently used in routine medical care. There are also endoscopic approaches, such as endocytoscopy, confocal laser endomicroscopy (CLE) and optical coherence tomography (OCT), that allow for a detailed examination of the intestinal surface and subsurface layer. Endocytoscopy or CLE gives detailed images of the ultra-magnified surface structure and microvessels, known as endoscopic histology, which may help improve the diagnostic performance [14–16]. However, the accuracy of these specific ultra-magnifying endoscopic diagnoses currently does not depend a complete one-to-one correspondence with the pathological findings, but rather it depends on the patterns observed between the endoscopic findings and the pathological diagnoses. Endocytoscopy or CLE may allow for more accurate endoscopic histology through a comparison with 3D reconstructed images. OCT is an imaging technology that captures structural images of biological tissue with high resolution in the micrometer scale using low-coherence light. OCT has been demonstrated to detect specialized transmural inflammation in IBD patients and has also been investigated for differentiating hyperplastic from adenomatous polyps in the colon [17, 18]. OCT angiography (OCTA) is another useful technique that enables the capture of real-time 3D microvasculature images by doppler OCT. OCTA has brought great influenced the diagnosis and management of disease in many fields of medicine, particularly ophthalmology [19]. However, as resolution of OCTA images is decreased due to the use of probe-type OCT in endoscopic OCTA, it was reported that this imaging modality mainly visualized not the superficial layer but subsurface vasculature with distance of >200 μm from the mucosal surface to microvessels [20]. On a comparison with cross-sectional pathological images, 3D-OCT microvasculature images may be more useful as a guide, as was noted in the current study. Because endoscopic OCTA mainly represents the subsurface structure, it may be difficult to compare the superficial microvasculature images created by NBI or BLI [21]. Thus, the 3D reconstructed pathological images of the current study may be suitable for comparing between cross-sectional pathological images and superficial microvasculature images, such as NBI and BLI. Regarding the detailed examination of the specialized microvascular system using 3D reconstruction techniques, such as those reported by Konerding or Skinner et al, it was shown that the microvascular structure differs according to the histological type [8, 10]. However, the microvasculature images in those previous studies were considered difficult to compare to endoscopic images because of the modality specializing in the presentation of the microvasculature and not in the detection of the superficial structure of grand orifices. In addition, a 3D structural analysis using serial block-face scanning electron microscopy (SBF-SEM) enabled the creation of 3D microvasculature images from resin-embedded specimen [22, 23]. It may be also difficult to match the 3D images obtained by SBF-SEM with the endoscopic images. However, prior to this report, no studies have detailed comparisons of endoscopically observed sites with 3D pathological reconstructed images. Thus, the present study focused on BLI, which is currently more useful for the analysis of the microvasculature, and compared images of magnifying BLI with pathological images using a 3D reconstruction technique to verify what BLI observes.

For the detailed comparison between endoscopic images and pathological images, several potential issues associated with specimen preparation and endoscopic observation should be considered. First, the specimen deforms and contracts during resection and the process of creating a pathological section. As the tissue contracts with formalin fixation, the blood vessel depth from the mucosal surface may be underestimated. Furthermore, this study requires that the surface layer be preserved. When a long time lapses after excision of a specimen, the surface layer peels off, making it difficult to evaluate the surface layer and the depth of vessels from the surface. Second, regarding endoscopic observation, the thickness of the mucosal tissue may vary depending on the air supply during endoscopic observation. This suggests that the distance from the surface layer to the blood vessel may change. And, the influence of the imaging angle of the endoscope during observation of the mucosal surface was not considered in this study. The optical path length to the blood vessel generally becomes long with a change in the angle. Especially in the case of soft and flat lesions, there were many differences from the endoscopic images due to the effect of stretching and fixing the specimen. Even if the endoscopic images after fixation of the specimen and the 3D reconstructed pathological images actually matched, there were lesions in which the images obtained at the endoscopic observation did not match the 3D reconstructed pathological images. Third, regarding the histological type, we reconstructed 3D images and evaluated the histological findings in homogeneous microstructural lesions with faint vessels (e.g., hyperplastic lesions or normal mucosa); however, it was extremely difficult to identify the specific microvessels or gland orifices for which accuracy of within a few micrometers would be required because the tissues were uniform. Fourth, regarding the slice direction, the vertically sliced 3D images were superior to the horizontally sliced 3D images in the superficial layer analysis, including providing a more detailed view of the pathological structures and depth of microvessels. It was very difficult to create the top view of 3D reconstructed images because the tissues were split when the mucosal surface was irregular in horizontal slice. Thus, in the horizontal 3D images, it was difficult to evaluate the superficial layer of the tumor for a comparison between 2D vertical pathological sections and endoscopic images.

The median microvasculature depth from the mucosal surface recognized on magnifying BLI was found to be 29.4 μm. On the other hand, a distance of >200 μm from the mucosal surface to microvessels was a cause of deviation on magnifying BLI. We detected microvessels on magnifying BLI in areas in which no vessels were observed on the pathological section. There was a horizontal gap of a few micrometers when magnifying BLI was compared to the pathological section. Even if the images matched completely, a gap of a few micrometers was present. Conversely, we also detected microvessels near the surface in the pathological section, in areas in which no vessels were observed by magnifying BLI. This gap occurred for the same reasons. When the above deviations were corrected, the new range from the mucosal surface to the visible vessels was 17.5–76.1 μm, and in the area in which vessels were not detected on magnifying BLI, the new range from the mucosal surface to the vessels was 77.3–382.7 μm. Considering the maximum depth of the visible vessels and the minimum depth of the invisible area, the boundary of the visible range was approximately 80 μm, which was much shallower than the theoretical distance reported previously. In a pioneering study using short wavelength light, Gono et al. measured the blood vessels on the mucosal surface of a human tongue using 5 narrow-band illuminations and showed that the detection of the superficial capillaries was markedly improved by exploiting light of around 415 nm [24, 25]. The optical penetration depth using a wavelength of 415nm±30 nm was 170 μm in this study. These authors also estimated that the superficial capillaries in colonic adenoma were about 150 μm from the mucosal surface. In the other animal experimentation using swine gastric mucosa, the contrast of superficial microvessels (10–100 μm deep from the mucosal surface) was the highest a short wavelength of 420 nm [26]. Although the penetration of the short wavelength light logically reached about 150–200 μm from the mucosal surface and the blood vessels of 10–200 μm in depth could be observed in these studies, it might be difficult to discuss the depth of blood vessels from the surface with a narrow scale like the one used in this study.

IEE using short wavelength light can visualize microvessels in the mucosal surface layer based on the characteristics of hemoglobin absorption. Thus, the area with erythrocytes appears brown on magnifying BLI. Conventionally, brownish areas have been thought to include capillaries and vessels of various degree of thickness [1]. We detected some brownish areas, including dark and light brownish areas on magnifying BLI and demonstrated that brownish areas were formed not only in dense blood vessels but also in the vasodilation and extravasation of erythrocytes. It is suggested that we can recognize most brown linear structures as blood vessels; however brownish areas can suggest various possibilities.

Several limitations associated with the present study warrant mention. This work was a preliminary study of a few colonic lesion cases. Although 3D reconstruction images can be generated from good condition resin-embedded specimens and 3D image analysis system such as Synapse VINCENT, ultimately these 3D images may not match the endoscopic findings. Thus, these cases showed the best results of the cases that we examined with one-to-one correspondence between the endoscopic images and 3D reconstructed pathological images that could be clearly understood. In addition, various biases including biases involving the site of the specimen, the method of observation and the range of observation were considered. During pathological specimen preparation, the specimen deforms and contracts during resection and the process of creating a pathological section. In addition, the resolution is limited by the thickness of the pathological slice. During the endoscopic observation, the parameter of the algorithms could not be set because the conditions were not constant due to effect of the air supply, observation angle or distance and intestinal peristalsis. When matching endoscopic images and 3D reconstruction images, the determination of the location depended on the characteristic endoscopic findings, such as the tumor shape. Furthermore, the 3D images in the present study could not be created in real-time, as in OCT and OCTA. Although this approach may have disadvantages in some cases and despite the abovementioned limitations findings from cases similar to analyzed in the present study, which represents the best case scenario, have impacted knowledge regarding the appearance of three-dimensional pathological features on two-dimensional endoscopy, and may lead to an accurate endoscopic diagnosis.

## Conclusion

The current study was the first report in which an endoscopic image was matched with a histopathological image using a 3D reconstruction model in human colonic tissues. This approach allowed us to investigate in detail the correspondence between the vascular pattern or surface pattern and the 3D-vascular network or glandular structure. In particular, we could measure the vascular depth from the mucosal surface of recognizable microvessels on magnifying BLI, and recognized that some brownish structures in magnifying BLI could be various structures including dense vessels, vessel expansions, corrupted vessels, extravasation of erythrocytes and crypt orifices. This approach may increase the overall understanding of endoscopic images and positively contribute to the making of more accurate endoscopic diagnoses.

## Supporting information

S1 Video 3D reconstructed histological images in the normal mucosa near the tumor. (MP4)

S2 Video 3D reconstructed histological images in colonic adenoma. (MP4)

S3 Video 3D reconstructed histological images in mucosal invasive adenocarcinoma (Fig 4–1). (MP4)

S4 Video 3D reconstructed histological images in mucosal invasive adenocarcinoma (Fig 4–2). (MP4)

S5 Video 3D reconstructed histological images in mucosal invasive adenocarcinoma (Fig 4–3). (MP4)

S6 Video 3D reconstructed histological images in mucosal invasive adenocarcinoma (Fig 4–4). (MP4)

S7 Video 3D reconstructed histological images in muscle invasive adenocarcinoma. (MP4)
